# Pre-Release Consumption of Methyl Eugenol Increases the Mating Competitiveness of Sterile Males of the Oriental Fruit Fly, *Bactrocera dorsalis*, in Large Field Enclosures

**DOI:** 10.1673/031.010.0801

**Published:** 2010-02-27

**Authors:** Todd E. Shelly, James Edu, Donald McInnis

**Affiliations:** ^1^ USDA-APHIS, 41-650 Ahiki Street, Waimanalo, HI 96795; ^2^ USDA-ARS, 2727 Woodlawn Avenue, Honolulu, HI 96822

**Keywords:** Diptera, Tephritidae, sterile insect technique, male lure, egg sterility, *Psidium guajava*, *Carica papaya*, *Malus domestica*

## Abstract

The sterile insect technique may be implemented to control populations of the oriental fruit fly, *Bactrocera dorsalis* (Hendel) (Diptera: Tephritidae), when environmental concerns preclude widespread use of chemical attractants or toxicants. The goal of the present study was to evaluate whether the mating competitiveness of sterile *B. dorsalis* males could be increased via pre-release feeding on methyl eugenol. Males of the oriental fruit fly are strongly attracted to this plant-borne compound, which they ingest and use in the synthesis of the sex pheromone. Previous studies conducted in the laboratory and small field-cages have shown that males given methyl eugenol produce a more attractive pheromone for females and have a higher mating success rate than males denied methyl eugenol. Here, levels of egg sterility were compared following the release of wild-like flies and either methyl eugenol-fed (treated) or methyl eugenol-deprived (control) sterile males in large field enclosures at four over flooding ratios ranging from 5:1 to 60:1 (sterile: wild-like males). Treated sterile males were fed methyl eugenol for 1–4 h (depending on the over flooding ratio tested) 3 d prior to release. Eggs were dissected from introduced fruits (apples), incubated in the laboratory, and scored for hatch rate. The effect of methyl eugenol was most pronounced at lower over flooding ratios. At the 5:1 and 10:1 over flooding ratios, the level of egg sterility observed for treated, sterile males was significantly greater than that observed for control, sterile males. In addition, the incidence of egg sterility reported for treated sterile males at these lower over flooding ratios was similar to that noted for treated or control sterile males at the 30:1 or 60:1 over flooding ratios. This latter result, in particular, suggests that pre-release feeding on methyl eugenol allows for a reduction in the number of sterile flies that are produced and released, thus increasing the cost-effectiveness of the sterile insect technique.

## Introduction

The sterile insect technique is an important component of many area-wide integrated pest management programs against tephritid fruit flies (Enkerlin 2005). Regardless of the strategic goal of the control program (e.g., eradication, suppression, etc.), the success of the sterile insect technique hinges, to a large extent, on the field performance of the mass-reared, sterilized, and released insects (Calkins et al. 1994). For certain fruit fly species, most notably the Mediterranean fruit fly, *Ceratitis capitata*, released males must have the capability to locate mating aggregations (leks), synthesize and broadcast attractive pheromones, and perform complex courtship displays involving olfactory, visual, and acoustic signals (Hendrichs et al. 2002; Robinson et al. 2002). Unfortunately, processes inherent in mass production, such as genetic drift and intense artificial selection under crowded rearing conditions, may result in the release of sterile male fruit flies that have low sexual competitiveness relative to their fertile, wild counterparts (Leppla 1989; Cayol 2000). Such poor field performance may, of course, greatly reduce the cost effectiveness of sterile insect programs.

There exists, therefore, a persistent and pressing need to improve the operating procedures used at fruit fly production and holding facilities in order to enhance the field performance of the released insects. In recent years, considerable research has focused on two potential avenues, both involving the adult stage, for improving the quality of sterile male fruit flies. First, numerous studies (*Ceratitis*: see Yuval et al. 2007 for review; *Bactrocera*: Shelly et al. 2005a; *Anastrepha*: Aluja et al. 2001) have evaluated the impact of nitrogenous supplements to standard sugar-based diets on male longevity and mating competitiveness. While such supplements often show a positive effect, this result is not universal, and inter-strain and inter-specific differences appear to exist. The second area of research has investigated the effect of chemical treatments - either topical application of juvenile hormone analogs (Teal et al. 2000, 2007), ingestion of botanical compounds (Papadopoulos et al. 2001; Shelly and Villalobos 2004), or exposure to airborne volatiles from such compounds (Shelly et al. 2007) - on the speed of male sexual maturation and/or male mating success. Most of the work regarding the practical application of “chemotherapy” has been limited to two species, *C. capitata* and the Caribbean fruit fly, *Anastrepha suspensa*, and the potential usefulness of a chemotherapeutic component in sterile insect programs against other important fruit fly species remains largely uninvestigated.

The use of chemicals, particularly male lures or parapheromones (Sivinski and Calkins 1986; Cunningham 1989), to manipulate behavior for management purposes has been well-documented for fruit flies in the genus *Bactrocera*, most notably *B. dorsalis* (Hendel) (Diptera: Tephritidae). Long known as a powerful male attractant for certain *Bactrocera* species (Howlett 1915; Steiner 1952), methyl eugenol is widely used to detect incipient infestations of *B. dorsalis* (Chambers et al. 1974; Jang and Light 1996). In addition, when mixed with a toxicant, methyl eugenol was used to eradicate island populations of this species in a procedure termed “male annihilation” (Steiner et al. 1965, 1970).

Although not frequently used against *B. dorsalis*, the sterile insect technique may be implemented in those instances where male annihilation alone is ineffective or where environmental concerns preclude widespread use of methyl eugenol, which may attract non-target species and pose a threat to native insect populations. For example, to reduce environmental hazards, Thailand and the International Atomic Energy Agency have implemented an area-wide integrated pest management effort, which involves the release of sterile males to control *B. dorsalis* (as well as *B. correcta*) in mango orchards (Orankanok et al. 2007).

Recent biochemical and behavioral studies indicate that methyl eugenol might be incorporated into such sterile insect programs as a means to increase the mating competiveness of released, sterile males. Methyl eugenol has been shown to function as a precursor for the male sex pheromone in *B. dorsalis* (Nishida et al. 1988, 1997) and to increase the attractiveness of the pheromone to females and, correspondingly, male mating success in trials conducted in laboratory cages or individually caged host trees (Shelly and Dewire 1994; Tan and Nishida 1996; Shelly and Nishida 2004). Like the Mediterranean fruit fly, *B. dorsalis* appears to exhibit a lek mating system, where males perch on leaf surfaces and emit a pheromone (dispersed by vigorous wing-fanning) to attract receptive females (Kobayashi et al. 1978). Females freely select their mates and, as courtship is absent, the pheromone signal appears critical in affecting male mating success (Shelly and Kaneshiro 1991). Additional studies (Shelly 1995; Shelly et al. 2000) demonstrated that the ingestion of methyl eugenol increased the mating competitiveness of mass-reared, sterile *B. dorsalis* males (relative to wild males), though these studies were again conducted in small cages in the laboratory. Interestingly, males fed methyl eugenol as larvae did not gain a mating advantage over non-fed males (Shelly and Nishida 2004).

The objective of the present study was to assess the effect of methyl eugenol consumption on the mating performance of sterile *B. dorsalis* males in more natural conditions. In particular, the level of egg sterility (evidence of successful mating by sterile males) was compared following release of wild flies and either methyl eugenol-fed (treated) sterile males or methyl eugenol-deprived (control) sterile males in large field enclosures. As described below, the enclosures contained multiple host plants and were much larger than the cages placed over single trees. While obviously not a complete substitute for open field tests, the large enclosures provided a more natural setting than the single-tree tents and, just as importantly, allowed for experimental manipulation of over flooding (sterile male: wild male) ratios and for replicated trials at the selected test ratios.

## Materials and Methods

### Study Insects

Mass-reared *B. dorsalis* males were from a genetic sexing strain based on a pupal color sexual dimorphism (males = brown; females = white). The strain was developed by McCombs and Saul (1995) and has been reared at the USDA-ARS laboratory, Honolulu, HI, at levels of 10,000–500,000 pupae per week. Eggs were collected in perforated tubes from large colony cages and placed on a wheat mill feed diet. Approximately 7 d later, the fully developed larvae “popped” into vermiculite, which was sifted 2 d later to collect pupae. To separate the sexes, pupae were passed through a high-speed, photoelectric sorting machine twice, and the male pupae were irradiated at 100 Gy under hypoxia 2 d prior to eclosion with a Gammacell 220 (Nordion Co., Canada) cobalt source.

Following irradiation, flies were held in different sized containers depending on the size of the release. For trials involving lower over flooding ratios (5:1 and 10:1), newly emerged adult males were placed in mesh screen cages (30 cm cubes with a cloth sleeve on one side). For trials involving higher over flooding ratios (30:1 and 60:1), irradiated pupae were placed in paper bags, which, in turn, were placed in plastic adult rearing containers (0.48 × 0.60 × 0.33 m; socalled PARC boxes). Sterile flies were held at 23–27 °C and 60–90% RH, and they received both natural and artificial light with a photoperiod of 12:12 (L:D). Food (a sugar-yeast hydrolysate mixture, 3:1 v:v) was placed in Petri dishes that were placed on the floor of the containers before introduction of the adult (small screen cages) or pupal (PARC boxes) males. In addition, sugar-agar gel and a honey-yeast hydrolysate paste (3:1, v:v) were applied to the top of the screen cages or the screened opening on the top of the storage boxes. For all containers, the sugar-agar gel was replaced every other day, and the food paste was applied daily. At release, the food dishes placed inside the containers invariably contained some residual material, indicating that the flies did not suffer a food shortage.

As described below, 200 wild-like males were released in all trials, which were conducted in two field enclosures concurrently, one involving control males and the other involving treated males. Thus, on any given day, we established equal numbers of containers for control and treated males. For trials involving 5:1 or 10:1 over flooding ratios, 250 sterile males were placed in each of 8 screen cages (4 control, 4 treated) or 16 screen cages (8 control, 8 treated), respectively. Surplus males were also maintained and used to replace dead individuals at the time of release. For trials involving 30:1 or 60:1 over flooding ratios, sterile males were placed in two storage boxes (1 control, 1 treated). For the 30:1 and 60:1 over flooding ratios, 180 and 360 ml of pupae (1 ml ≈ 40 pupae), respectively, were distributed evenly among six bags per storage box. The number of pupae placed in the boxes exceeded the designated over flooding ratio by 20% (≈ 7,200 and 14,400 pupae for 30:1 and 60:1, respectively) to compensate for mortality and developmental abnormalities that prevented flight.

Wild-like flies were from a laboratory colony started with 400–600 adults reared from field-collected guava fruits, *Psidium guajava* L. (Myrtales: Myrtaceae). The colony was housed in a screen cage (0.6 × 0.6 × 1.2 m) with superabundant food (the sugar-yeast hydrolysate mixture), water, and ripe papayas, *Carica papaya* L. (Brassicales: Caricaceae) were provided periodically for oviposition. Infested fruits were held over vermiculite for about three weeks, and pupae were then collected by sifting the vermiculite. Adults were separated by sex within 24 h of emergence and were provided the sugar-yeast hydrolysate diet and water. The wild-like flies were held under the same environmental conditions as the sterile males. When tested, the wild-like flies were 3–7 generations removed from the wild.

### Exposure to Methyl Eugenol

For all trials, treated sterile males were given access to methyl eugenol 3 d before release, while control sterile males and wild-like males were not supplied with the chemical. Previous work (Shelly and Dewire 1994) showed that consumption of methyl eugenol boosted male mating success over intervals lasting from 1–35 d post-feeding. Methyl eugenol (provided by FarmaTech International, www.farmatech.com) was applied to cotton wicks resting in aluminum foil-lined Petri dishes (one wick per dish), which were then placed directly in the holding containers. For trials involving the lower over flooding ratios, we applied 0.25 ml of methyl eugenol to a single cotton wick (1 cm diameter, 7.5 cm long), which was then placed in individual screen cages for 1 h. For trials involving the higher over flooding ratios, we applied 1 ml of methyl eugenol to each of three 3 wicks (1 cm diameter; 15 cm long), which were then placed in individual storage boxes for 2 (30:1) or 4 (60:1) h (wicks were introduced and removed through a hole drilled in the side of the storage boxes plugged with a foam stopper). Thus, while the per capita dose of methyl eugenol was lower in the storage boxes than the screen cages, the exposure interval was longer in the storage boxes to increase access to the chemical. Exposure to methyl eugenol was initiated between 0900-1000 hrs and conducted in a room isolated from control males, although prior work (Shelly and Dewire 1994) demonstrated that exposure to the aroma alone (i.e., without ingestion) had no effect on male mating success. When given access to methyl eugenol, the treated males were 6–7 d old (the age at which males of this strain typically attain sexual maturity, TE Shelly, unpublished data).

### Methyl Eugenol Consumption and Male Mortality

Steiner (1952) reported that, given free access to methyl eugenol, *B. dorsalis* males will “kill themselves with overindulgence.” To determine whether the protocol for pre-release feeding on methyl eugenol influenced male mortality, 30 screen cages (30 cm cubes with cloth sleeve on one side) were set up each containing 20 sterile males (8 d old) and ample food and water. On the same day, methyl eugenol (0.25 ml applied to a cotton wick resting in a Petri dish) was introduced for 1 h to 15 of the cages. Starting the next day, dead males were removed on a daily basis, and after 1 wk, the total number of deaths between cages that did and did not receive methyl eugenol were compared. Cages were held under the environmental conditions described above.

### Field Trials

Trials were conducted in four nylon-screen enclosures (16 m long × 6 m wide × 2.5 m high) set up in a guava orchard (*P. guajava*) in Waimanalo, Oahu (elevation 20 m). The tents, which were parallel to one another and separated by 5 m, contained 10–15 guava trees and were covered with shade screen to reduce insolation. As described below, trials lasted 5 d, and treated and control males were tested concurrently (i.e., one treatment per enclosure). Enclosure pairings were the same over the entire study, resulting in a use/nonuse schedule that alternated weekly for each pair of enclosures. Within each pair of enclosures, the type of sterile male released in a given enclosure (i.e. control or treated) was alternated between successive replicates. Trials were conducted between September 2005 and April 2006. Daily minimum and maximum air temperatures were recorded over the entire study at a weather station situated ca. 100 m from the enclosures.

The same schedule was followed for all trials. On day 1, food and water were introduced, and the flies were released. Food (the sugar-yeast hydrolysate mixture) was presented in Petri dishes held in Jackson traps (lacking sticky inserts) suspended 1.5–2.5 m above ground from tree branches at four evenly spaced locations. At each of these sites, water was provided in a covered plastic cup (100 ml volume with an emerging cotton wick) held within a Jackson trap. The wires suspending the resource-laden Jackson traps from branches were coated with Tanglefoot® (Tanglefoot Company, www.tanglefoot.com) to exclude ants. Food and water were not replaced during a trial. Following the placement of food and water, the flies were released in the center of the enclosures. All releases were performed between 1000 and 1100 hrs. Mating activity in *B. dorsalis* is restricted to a narrow time window immediately preceding and following sunset (Roan et al. 1954, Arakaki et al. 1984).

On day 4, 15 Granny Smith apples, *Malus domestica* (Borkh) (Rosales: Rosaceae), were placed in the enclosures at 1000 hrs for oviposition. Apples were suspended 1.5–2.5 m above ground by piercing the fruit with a nail and connecting the nail to a branch with wire. In addition, to simulate wounds and facilitate oviposition, 10–15 shallow holes were made in each apple using a toothpick. Tanglefoot® was applied to the wire to exclude ants. The apples served as the only available oviposition resource as the guava fruits were removed before the trials. On day 5, the apples were collected, marking the end of the trial (food and water were also removed at this time).

For each trial, the egg hatch of wild-like females mated exclusively to wild-like males in a field cage over a single guava tree adjacent to the large enclosures was also measured. Two hundred individuals of each sex were introduced on day 1, and two apples were introduced on day 2 for a 24 h period.

Upon collection, apples were returned to the laboratory, and eggs were removed using a scalpel and a fine forceps under a dissecting microscope. Eggs were placed on moistened blotter paper in covered Petri dishes and then incubated at 27 °C for 72 h. Hatch was then determined by re-examining the eggs under a dissecting microscope.

Seven replicates were conducted for each over flooding ratio, where a replicate consisted of treated and control males placed concurrently, but separately, in the large enclosures (with associated wild-like flies) and wild-like flies exclusively placed in the single, caged guava tree. At the end of a trial, several Jackson traps baited with food and containing sticky inserts were suspended in the guava trees and left in place until the next trial to remove the remaining, previously released flies.

### Fried's Competitiveness Index (C)

For each replicate, Fried's (1971) competitiveness index (C) was computed to compare the performance of control and treated sterile males vs. wild-like males, where C = (W/S) × [(H_w_ - H_c_)/(H_c_ - H_s_)], with W = number of wild-like males released, S = number of sterile males released, Hw = percentage of egg hatch from wild-like females following mating with wild-like males (as determined from the single caged guava tree), Hc = percentage of egg hatch from wild-like females in the test enclosure, and Hs = percentage of egg hatch from wild-like females following mating with sterile males (= 0.2%, DOM, unpublished data).

### Daily Fecundity

To estimate the number of females depositing eggs in the apples provided in the field enclosures, daily egg production was estimated in the laboratory. Using wild-like individuals exclusively, mating pairs were collected from Plexiglas cages (30 × 30 × 40 cm) and, the following morning, the mated females were transferred to a screen cage containing food and water but no oviposition substrate. Two days after mating, females were placed singly in screen cages (30 cm cubes) along with an apple (containing puncture wounds as described above) at 0800 hrs and removed at 1800 hrs. Apples were kept in a refrigerator (10 – 12 °C) overnight, and egg counts were made the following morning. Because trials did not span the entire dawn-dusk period (approximately 12 h), the estimates of daily fecundity are likely conservative.

### Statistical Analysis

Male mortality in cages that did or did not receive methyl eugenol was compared using a t-test. Values for total egg abundance (normalized using a log_10_ transformation), egg sterility (arc sine transformed proportions of unhatched eggs/total eggs), and Fried's Competitiveness Index (arc sine transformed indices) were analyzed using two-way ANOVA with over flooding ratio and male treatment category (treated or control) as the factors. When significant variation was detected, the Tukey multiple comparisons test (critical value q) was used to identify pair wise differences among groups (test statistic q, with df = 48 [ANOVA error df] and k [number of groups] = 8 for all comparisons). Mean values ± 1 SE are presented.

Although daily maximum temperatures varied significantly among the time intervals when the four over flooding ratios were investigated (H = 71.9, df = 3, P < 0.001, Kruskal-Wallis test), temperature was excluded as a factor in the analyses for two reasons. First, the temperature differences, while consistent, were quite small. Among the study periods, the mean daily maximum temperatures ranged only between 25.7 – 29.1 °C. In addition, no significant correlation was found between the mean daily maximum temperature during a replicate and (i) the total number of eggs collected, (ii) the total number of apples containing eggs, or (iii) the proportion of sterile eggs when considering data from treated and control groups separately or collectively within or between over flooding ratios (P > 0.05 in all cases, Spearman rank correlation).

## Results

### Methyl Eugenol Consumption and Male Mortality

There was no significant difference in the mean number of male deaths recorded for cages that did (2.1 ± 0.4) or did not (2.2 ± 0.4) receive methyl eugenol (t = 0.1, df = 28, P = 0.91).

### Total Egg Production

**Figure 1:**
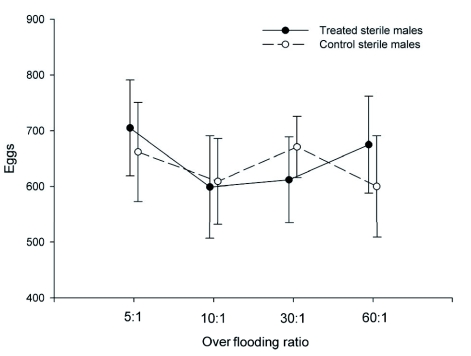
Total number of eggs collected from apples suspended in field enclosures with treated (provided methyl eugenol) or control (not provided methyl eugenol) sterile males of *Bactrocera dorsalis* at the four over flooding ratios (sterile: wild-like males) tested. Symbols represent means (± SE; n = 7). High quality figures are available online.

The total number of eggs collected per replicate varied independently of over flooding ratio (F_3, 48_ = 1.34, P = 0.27) and male treatment category (F_1, 48_ = 1.00, P = 0.32). The interaction term between main effects was also not significant (F_3, 48_ = 0.66, P = 0.58). Including all combinations of over flooding ratio and sterile male treatment category, 599–705 total eggs were collected, on average, per replicate (Fig. 1). On average, eggs were found in 8.9 (of a possible 15) apples per replicate (± 0.3, range: 5–11, n = 56).

### Daily Fecundity

Egg production was monitored in the laboratory for 41 females over a 10-h period. Approximately 30% (12/41) of the females laid no eggs at all, while the remaining females laid between 7–64 eggs with an average output of 26.2 eggs (± 3.3; n = 29). Based on this average daily output and the average total numbers of eggs collected per replicate, it was estimated that, on average, 23–27 females deposited eggs in the apples during a given replicate.

### Egg Sterility and Fried's Competitiveness Index

**Figure 2:**
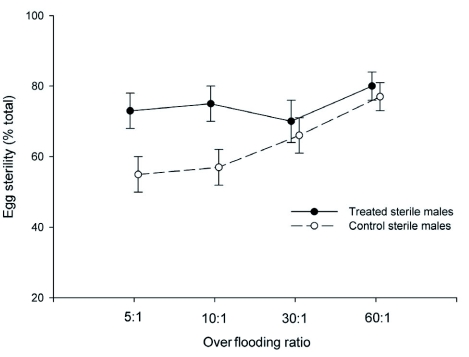
Relative number of sterile (unhatched) eggs collected (% total eggs collected) for treated (provided methyl eugenol) and control (not provided methyl eugenol) sterile males of *Bactrocera dorsalis* at the four over flooding ratios (sterile: wild-like males) tested. Symbols represent means (± SE; n = 7). High quality figures are available online.

Levels of egg sterility varied significantly with over flooding ratio (F_3, 48_ = 3.8, P = 0.02) and male treatment category (F_1, 48_ = 12.8, P < 0.001; Fig. 2). The interaction term was not significant (F_3, 48_ = 0.7, P = 0.55). The effect of over flooding ratio on egg sterility levels differed between treated and contol males. For treated males, egg sterility levels were fairly consistent across the different over flooding ratios, with average levels varying only between 73%–80%, and correspondingly, there were no significant differences in pair-wise comparisons (P > 0.05 in all cases). In contrast, for control males egg sterility varied markedly with over flooding ratio, increasing from 55% at the 5:1 over flooding ratio to 77% at the 60:1. For control males, significant differences in egg sterility were detected between the 60:1 over flooding ratio and the 5:1 (q = 4.2, P = 0.02) and 10:1 (q = 3.9, P = 0.04) over flooding ratios, respectively. For control males, the average sterility level observed at the 60:1 ratio was higher than that noted at the 30:1 ratio (77% versus 66%, respectively), but this difference was not significant (q = 3.4, P = 0.09).

Consistent with these trends, differences in egg sterility between treated and control males were more pronounced at lower rather than higher over flooding ratios. Egg sterility levels were approximately 33% greater for treated than control males at both the 5:1 over flooding ratio (73% versus 55%, q = 4.6, P = 0.04) and the 10:1 over flooding ratio (75% versus 57%, q = 4.8, P = 0.03). In contrast, the egg sterility values were only slightly higher for treated males at the higher over flooding ratios, and these differences were not statistically significant (30:1: 70% versus 66%, q = 2.4, P = 0.70; 60:1: 80% versus 77%, q = 2.1, P = 0.81).

**Figure 3:**
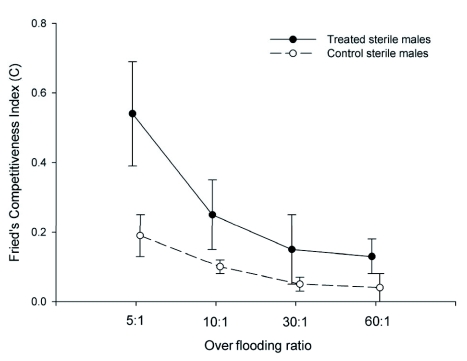
Fried's Competitiveness Index (C) for treated (provided methyl eugenol) and control (not provided methyl eugenol) sterile males of *Bactrocera dorsalis* at the four over flooding ratios (sterile: wild-like males) tested. Symbols represent means ± SE; n = 7). High quality figures are available online.

As with egg sterility levels, values of Fried's Competitiveness Index (C) varied significantly with over flooding ratio (F_3, 48_ = 5.5, P = 0.002) and male treatment category (F_1, 48_ = 10.1, P = 0.003; Fig. 3). The interaction term was not significant (F_3, 48_ = 1.3, P = 0.28). Competitiveness values reflected the relationships described above between over flooding ratio and egg sterility (Fig. 3). For treated males, the relatively constant level of egg sterility recorded over the different over flooding ratios resulted in decreasing C values with increasing over flooding ratio: the C value for the 5:1 over flooding ratio was significantly different from C values obtained at 30:1 (q = 5.1, P = 0.02) and 60:1 (q = 6.5, P < 0.001). No significant differences were detected between C values for the 5:1 and 10:1 over flooding ratios (q = 3.8, P = 0.15) or among C values recorded for over flooding ratios ≥ 10:1 (P > 0.05 in all cases). A similar decrease in competitiveness values was observed for control males with increasing over flooding ratios, but there were no significant differences detected among C values for the different over flooding ratios (P > 0.05 in all cases). This result derived from the increased egg sterility noted at the higher over flooding ratios (Fig. 2), which counterbalanced the effect of increasing over flooding ratio in the computation of C values.

Reflecting the convergence in egg sterility levels with increasing over flooding ratio (Fig. 2), competitiveness values for treated sterile and control sterile males differed significantly only at the lowest (5:1) over flooding ratio (q = 6.9, P < 0.001). Competitiveness indices were greater for treated sterile males than control sterile males at all higher over flooding ratios as well, but these differences were not statistically significant (P > 0.05 in all cases).

## Discussion

Pre-release feeding on methyl eugenol by sterile males of *B. dorsalis* resulted in increased levels of egg sterility at all over flooding ratios tested, although significant differences between methyl eugenol-fed and methyl eugenol-deprived sterile males were observed only for the lower ratios. Competitiveness indices computed for treated and control sterile males displayed the same trend. The level of egg sterility achieved by treated males at the lowest over flooding ratio tested (5:1) was similar to that observed for treated or control males at the highest ratios tested (30:1 or 60:1), indicating that pre-release feeding on methyl eugenol could allow reduction in the number of sterile males produced and released in control programs.

Although these data suggest a benefit to pre-release feeding on methyl eugenol, several factors potentially confound this interpretation. First, as control programs may release sterile males on a weekly or bi-weekly basis, these tests (lasting only 5 d) may not have detected any decrease over time in the mating competitiveness of sterile males, due to a possible “wearing off of the methyl eugenol-mediated mating enhancement. As noted above, a laboratory study (Shelly and Dewire 1994) showed increased mating performance for as long as 35 d after consumption of methyl eugenol, but the dose used was extremely high (1.5 ml of methyl eugenol per 40–60 males for 2 h), and it is not known if the doses employed in the current study confer a similar long-lasting benefit. Second, although no adverse effect of methyl eugenol feeding on male survival was found, these trials were conducted in the laboratory, and their applicability to the field is unknown. However, these data were important in revealing that there is no immediate toxic consequence of methyl eugenol ingestion.

In addition, although the large enclosures provided a semi-natural environment, the experiments nonetheless precluded dispersal as an influence on male mating competitiveness. If, for some reason, ingestion of methyl eugenol impairs flight propensity or ability, then treated sterile males may be less likely to locate preferred habitat, including optimal mating (lek) sites. Previous work (Shelly 1994) showed that methyl eugenol-fed males of *B. dorsalis*, which were subsequently marked and released in the field, were, in fact, less likely to be captured in methyl eugenol-baited traps than were methyl eugenol-deprived males. However, rather than indicating heightened mortality associated with methyl eugneol ingestion, the decreased trap capture apparently reflected decreased responsiveness of once-fed males to methyl eugenol. In other words, assuming dispersal ability is not reduced, pre-release exposure to methyl eugenol may serve to reduce the attraction of sterile male to methyl eugenol-baited traps, allowing concurrent implementation of the sterile insect technique and male annihilation.

As a final consideration, the fact that the wild-like males were not given methyl eugenol in any of the trials implies that the mating competitiveness of treated sterile males was overestimated. In the field, wild males will, of course, seek out sources of methyl eugenol and thereby enhance their mating ability. Thus, denying the wild-like males access to this chemical essentially “handicapped” them relative to treated sterile males. Despite this bias, pre-release exposure to methyl eugenol should prove beneficial, because it eliminates the “need” for sterile males to locate natural sources of the chemical in the environment (thereby eliminating time and energy costs associated with searching) and guarantees that sterile males gain the full benefits derived from methyl eugenol ingestion.

The present results were comparable to those obtained previously for the Mediterranean fruit fly. In that species, laboratory (Papadopoulos et al. 2006) and field-cage trials (Shelly et al. 2004) showed that sterile males exposed to the aroma of ginger root oil (*Zingiber officinale*) competed more successfully against wild males for matings with wild females than non-exposed sterile males. A subsequent experiment (Shelly et al. 2005b) using a similar protocol and the same large enclosures described in the present study showed that levels of egg sterility were significantly higher for ginger root oil-exposed sterile males than non-exposed sterile males. As with methyl eugenol, exposure to ginger root oil had its greatest impact at the lowest over flooding ratio tested, and the magnitude of the effect was similar between the two substances. At the 5:1 over flooding ratio, the level of egg sterility achieved by methyl eugenol-fed, sterile males of *B. dorsalis* was 33% greater than that observed for non-fed, sterile males (73% versus 55%, respectively), and in *C. capitata* egg sterility associated with ginger root oil-exposed, sterile males was 28% higher than that observed for non-exposed, sterile males (86% versus 67%, respectively). In contrast, at a 60:1 over flooding ratio, the proportional increase in egg sterility observed for treated, sterile males over control, sterile males was only 4% for *B. dorsalis* (80% versus 77%, respectively) and 7% for *C. capitata* (96% versus 90%, respectively). Although the trends were similar between species, exposure to ginger root oil had a more pronounced effect than feeding on methyl eugenol: levels of egg sterility and Fried's competitiveness index were significantly higher for treated than control males of *C. capitata* at all over flooding ratios tested, not only at lower over flooding ratios as observed in *B. dorsalis*.

Despite this difference, data from both species revealed the same underlying trend: treated sterile males released at low over flooding ratios achieved the same levels of egg sterility as control males released at high over flooding ratios. As noted above, this finding implies that pre-release treatment might increase the cost-effectiveness of the sterile insect technique by reducing the quantity of sterile flies required to achieve programmatic goals. Based on the protocol used for exposing sterile males at the 60:1 over flooding ratio (3 ml of methyl eugenol per 14,400 flies), pre-release feeding would cost approximately $5 per million males (at $22 per liter of methyl eugenol, J. Knapp, personal communication). Assuming production costs for *B. dorsalis* are similar to those for *C. capitata* ($100–$200 per million male pupae), methyl eugenol treatment would add only 2–5% to the production cost of the flies. Although data from open field releases are still lacking, the present findings strongly suggest that the increased mating competitiveness of sterile males justifies the small additional cost of implementing pre-release feeding on methyl eugenol.
